# Categorical-dimensional approach to diagnostic of schizotypal disorder

**DOI:** 10.1192/j.eurpsy.2021.1704

**Published:** 2021-08-13

**Authors:** A. Kotsiubinskii, Y. Isaenko, S. Kusnetsova

**Affiliations:** Biopsychosocial Therapy Department, The Saint-Petersburg National Medical Research Сentre n.a. V.M. Bekhterev, Saint-Petersburg, Russian Federation

**Keywords:** Categorical, dimensional, approach, schizotypal disorder

## Abstract

**Introduction:**

The relevance of this research is determined by the fact that an important scientific task of the modern clinical classification of mental disorders is the productive combination of the most valuable for the practical use of categorial and dimensional (in terms of the weight and depth of each dimensia) of the characteristics in in a particular clinical picture of a disease.

**Objectives:**

The goal of the research is to validate the new categorical-dimensional criteria necessary for the verification of schizotypal disorder.

**Methods:**

The information base of the research included medical data on 150 patients with schizotypal disorder. Categorical characteristic used according to the systematics of schizotypal disorder (Kotsiubinskii A.P, 2018) published in the National Guide «Psychiatry», which includes the following syndromes: obsessive-phobic, dysmorphophobic, non-delusional hypochondria, heboid, histrionophoric, impulsive-dysfunctional, schizoaffective, dissociative-disintegrative, autistic, dismotivative, amotivative. Our systematics was used with following demensia: positive, affective, negative, cognitive, disordered behavior, dissociative and coenestesipatic. Guided by the principle of five-level representation of each dimensia (from «0» to «4») in accordance with DSM-V and the informative systematics of dimensia was developed with each of dimensia also has rate from «0» to «4».

**Results:**

This diagnostic approach made it possible to correlate the categorial and dimensional characteristics, both to each other and to the criteria of the condition of the patients’ state with the prototype of schizotypal disorder (in the range of «1» to «5»).

**Conclusions:**

This has made it possible to more accurately diagnose non-psychotic forms of mental illness, in particular: differentiate schizotypal disorder «sui generis» and schizotypal personality disorder.

**Disclosure:**

No significant relationships.

